# Autonomic responses during gambling: the effect of outcome type and sex in a large community sample of young adults

**DOI:** 10.1192/j.eurpsy.2022.764

**Published:** 2022-09-01

**Authors:** C. Hultman, S. Vadlin, M. Rehn, K. Nilsson, C. Åslund

**Affiliations:** Center for clinical research, Region Västmanland, Västerås, Department Of Public Health And Caring Science, Uppsala University, Bmc, Uppsala, Västerås, Sweden

**Keywords:** Gambling, near-miss, autonomic responses, sex differences

## Abstract

**Introduction:**

Autonomic arousal is believed to be an underlying reinforcer for problematic gambling behavior. Theories suggests that near-misses (outcomes falling just short of a true win) are structural characteristics affecting emotion and motivation while increasing gambling persistence.

**Objectives:**

Psychophysiological responses to different outcomes in gambling were investigated in a community-based sample of young adults. Furthermore, sex differences in responses to different gambling outcomes were investigated.

**Methods:**

Young adults (n=270) performed a simplified virtual slot machine producing wins, two types of near-misses (before/after payline) and full-misses, with simultaneous measurements of heart rate (HR) and skin conductance responses (SCR). Self-reports of perceived chance of winning, pleasure and motivation to play were given by the participants on each trial.

**Results:**

Near-misses were associated with the largest HR acceleration compared to wins and full-misses, and larger HR deceleration and SCRs compared to full-misses. Differential autonomic and subjective reports were observed for near-misses subtypes, suggesting that near-misses are processed differently depending on their position before or after payline. Females showed larger SCR responses and increased motivation following wins compared to males.

**Conclusions:**

Slot machine gambling outcomes elicit differential physiological and subjective responses in young adults. Specifically, near-misses produce larger autonomic responses compared to regular full-misses. However, near-misses are complex, multifaceted events producing various emotional responses depending on their characterization. Males and females respond differently to wins, highlighting the importance of considering sex differences in experimental research on autonomic responses in gambling.

**Disclosure:**

No significant relationships.

